# Genome-based reclassification of Streptosporangium sandarakinum Kämpfer et al. 2013 as a later heterotypic synonym of Streptosporangium nondiastaticum Nonomura and Ohara 1969 (Approved Lists 1980)

**DOI:** 10.1099/ijsem.0.007359

**Published:** 2026-11-12

**Authors:** Adrianna Cruz, Richard W. McLaughlin, Mostafa S. Elshahed, Paul A. Lawson, Samuel L. Miller

**Affiliations:** 1 School of Liberal Arts and Sciences, Gateway Technical College, Kenosha, WI 53144, USA; 2 Department of Microbiology and Molecular Genetics, Oklahoma State University, Stillwater, OK 74074, USA; 3 School of Biological Sciences, University of Oklahoma, Norman, OK 73019, USA

**Keywords:** heterotypic synonym, *Streptosporangium*, *Streptosporangium nondiastaticum*, *Streptosporangium sandarakinum*

## Abstract

A comparison of 16S rRNA gene sequences between *Streptosporangium sandarakinum* GW-12028^T^ and *Streptosporangium nondiastaticum* IFO 13990^T^ revealed a sequence similarity of 98.8%. The genus *Streptosporangium* includes 33 species with validly published names. The type strains *S. sandarakinum* GW-12028^T^ (=DSM 45763^T^=LMG 27062^T^) and *S. nondiastaticum* IFO 13990^T^ (=ATCC 27101^T^=DSM 43848^T^=JCM 3114^T^=KCTC 9434^T^=NBRC 13990^T^=VKM Ac-1299^T^) were both isolated from soil. Whole-genome data were used to clarify the taxonomic assignment of these two closely related *Streptosporangium* species. Overall genomic relatedness index analyses indicated that *S. sandarakinum* GW-12028^T^ and *S. nondiastaticum* IFO 13990^T^ shared average nucleotide identity and digital DNA–DNA hybridization values >95% and >70%, respectively, exceeding the thresholds for bacterial species delineation. Additionally, *S. sandarakinum* GW-12028^T^ and *S. nondiastaticum* IFO 13990^T^ displayed near-identical phenotypic and chemotaxonomic profiles. Based on the 16S rRNA gene phylogenetic tree and the core-genome phylogenomic trees, *S. sandarakinum* GW-12028^T^ and *S. nondiastaticum* IFO 13990^T^ phylogenetically form a separate, tight cluster apart from other *Streptosporangium* species. Based on this combined evidence, *S. sandarakinum* Kämpfer *et al*. 2013 is proposed as a later heterotypic synonym of *S. nondiastaticum* Nonomura and Ohara 1969.

## Introduction

The genus *Streptosporangium* was originally proposed by Couch in 1955 [1], in which *Streptosporangium roseum* is the type species. Members of this genus produce aerial mycelia, spherical sporangia and non-motile sporangiospores. To date, *Streptosporangium* includes 33 species with validly published names (https://lpsn.dsmz.de/genus/streptosporangium, April 2026). Members of this genus have been isolated from different environments, including volcanic ash [2], lake sediments [3], roots [4], lichens [5], millipedes [6] and soils [7, 8]. Both *Streptosporangium sandarakinum* GW-12028^T^ and *Streptosporangium nondiastaticum* IFO 13990^T^ were originally isolated from soil and are Gram-stain-positive, aerobic bacteria that produce sporangia, show filamentous growth and display similar chemotaxonomic profiles [7–9]. Highly conserved phenotypic characteristics were observed, including the utilization of d-glucose, l-arabinose, d-galactose, d-fructose, d-mannose, d-mannitol and d-xylose, as well as liquefaction of gelatin and melanin production, for *S. sandarakinum* GW-12028^T^ and *S. nondiastaticum* IFO 13990^T^. Minor physiological differences between the two strains include sorbitol and melibiose utilization, the degradation of cellulose, nitrate reduction and the hydrolysis of l-malate [7]. Chemotaxonomically, the respiratory quinone profiles of *S. sandarakinum* GW-12028^T^ and *S. nondiastaticum* IFO 13990^T^ exhibit significant similarity, consistent with their close phylogenetic relationship. Both strains predominantly utilize menaquinone-9 (MK-9) and its hydrogenated derivatives as their primary electron carriers. Specifically, *S. sandarakinum* GW-12028^T^ is characterized by a major quinone system consisting of MK-9, MK-9(H_2_) and MK-9(H_4_) [7]. This aligns closely with *S. nondiastaticum* IFO 13990^T^, which similarly possesses MK-9, MK-9(H_2_), MK-9(H_4_) [specifically the MK-9(III, VIII-H_4_) isomer] and MK-9(H_6_) [9].

Upon comparison, the 16S rRNA gene sequences of *S. sandarakinum* GW-12028^T^ (JX977118) and *S. nondiastaticum* IFO 13990^T^ (X89945) were found to be 99.8% identical (Table 1), exceeding the 98.7% threshold value used in the assignment of strains to a species [10]. The EzBioCloud server [11] was then used to confirm their apparent close relationship. The 16S rRNA gene sequences of other members of the genus *Streptosporangium* with validly published names were downloaded, aligned using ClustalW [12], and a phylogenetic analysis was constructed in mega12 [13], using the maximum-likelihood (ML) algorithm [14] (Fig. 1). Genetic distances were then calculated using the Kimura two-parameter model [15], and bootstrap values were calculated using the standard 1,000 replications [16]. The 16S rRNA gene tree obtained confirmed the nearly identical relationship between *S. sandarakinum* GW-12028^T^ and *S. nondiastaticum* IFO 13990^T^ (Fig. 1).

**Fig. 1.**
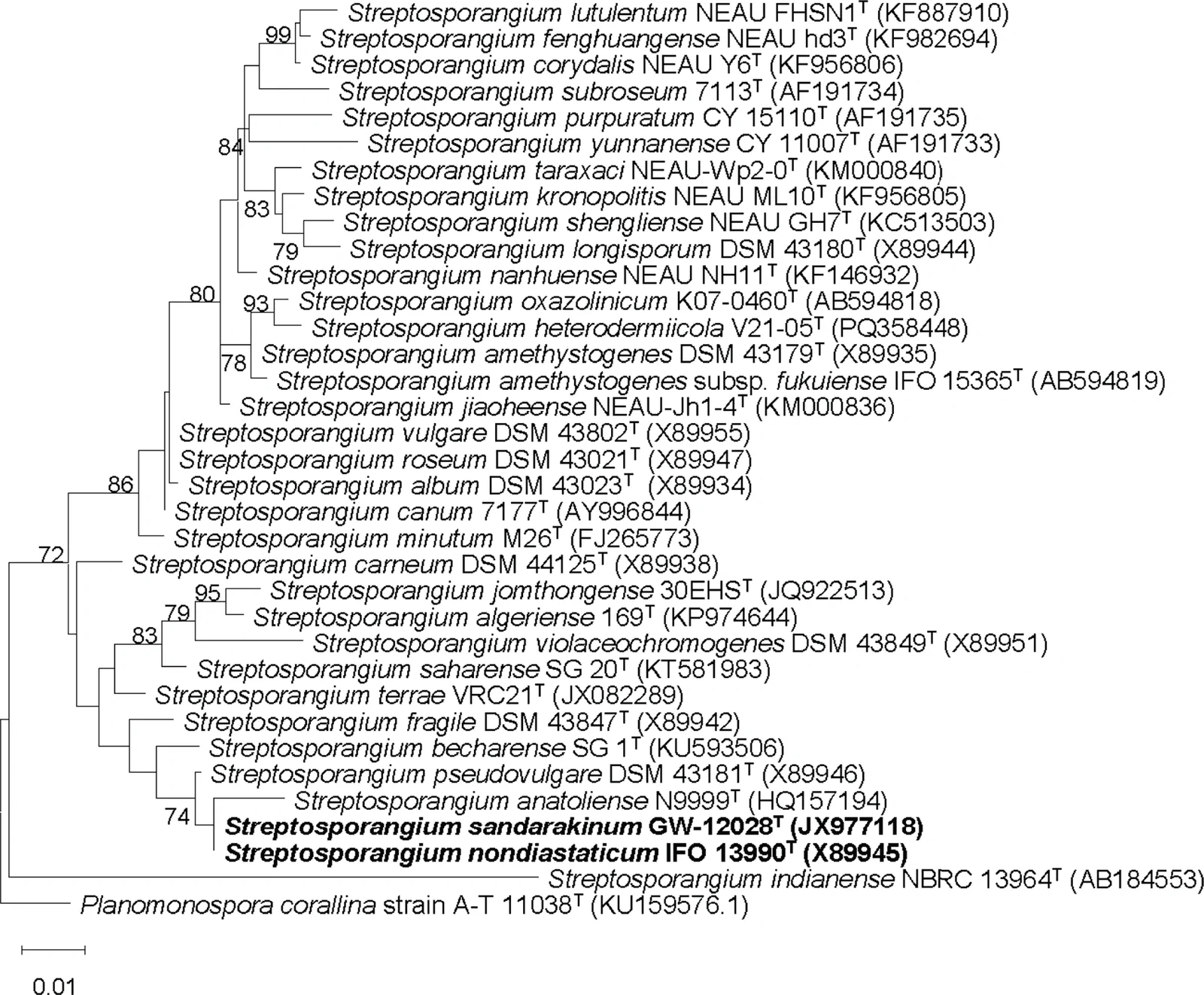
Phylogenetic tree based on 16S rRNA gene sequences (1,372 bp) of *S. sandarakinum* GW-12028^T^ and *S. nondiastaticum* IFO 13990^T^ and their nearest phylogenetic relatives. The tree was constructed using the ML method. Bootstrap values, expressed as percentages of 1,000 replications, are given at branching points. Database accession numbers are given in parentheses. Bar indicates the substitutions per nucleotide position, 0.01.

**Table 1.** Pairwise comparison of 16S rRNA gene similarity and overall genomic relatedness indices between *S. nondiastaticum* IFO 13990^T^, *S. sandarakinum* GW-12028^T^ and the type strains of their close relatives

16S rRNA gene similarity (%)				
**Taxa**	**16S accession**	***S. nondiastaticum***	***S. sandarakinum***	
***S. nondiastaticum***	**X89945**	**100.0**	**99.8**	
***S. sandarakinum***	**JX977118**	**99.8**	**100.0**	
*S. pseudovulgare*	X89946	98.3	98.0	
*S. minutum*	FJ265773	97.8	97.6	
*S. canum*	AY996844	97.6	97.5	
**dDDH (%**)				
**Taxa**	**WGS accession**	***S. nondiastaticum***	***S. sandarakinum***	
***S. nondiastaticum***	**GCA_039535295.1**	**100.0**	**81.5**	
***S. sandarakinum***	**GCA_013409935.1**	**81.5**	**100.0**	
*S. pseudovulgare*	GCA_014648055.1	66.3	66.2	
*S. minutum*	GCA_002149035.1	29.2	29.1	
*S. canum*	GCA_900113865.1	29.1	29.0	
**ANI (%)**				
**Taxa**	**WGS accession**	***S. nondiastaticum***	***S. sandarakinum***	
***S. nondiastaticum***	**GCA_039535295.1**	**100.0**	**98.2**	
***S. sandarakinum***	**GCA_013409935.1**	**98.2**	**100.0**	
*S. pseudovulgare*	GCA_014648055.1	96.6	96.6	
*S. minutum*	GCA_002149035.1	85.4	85.4	
*S. canum*	GCA_900113865.1	85.5	85.5	

Based on the high 16S rRNA gene sequence similarity values observed, further analyses were conducted for both strains to establish a more robust species-level taxonomic differentiation and clarify their taxonomic relationship. Since genomic sequences are publicly available for *S. sandarakinum* GW-12028^T^ (GenBank accession number GCA_013409935.1) and *S. nondiastaticum* IFO 13990^T^ (GCA_039535295.1), the phylogenetic relationship between the two species was further explored. The average nucleotide identity (ANI) [17] and digital DNA–DNA hybridization (dDDH) [18] between strains GW-12028^T^ and IFO 13990^T^, as well as other close relatives, were calculated. An orthoANI [19] value of 98.2% was determined from their genomic sequences. This exceeds the currently accepted species cut-off values for ANI (95.0%–96.0%) [17]. An ANI value of 96.6% was obtained when *S. sandarakinum* GW-12028^T^ and *S. nondiastaticum* IFO 13990^T^ were compared to *Streptosporangium pseudovulgare* DSM 43181^T^, respectively. Further, to ensure no further heterotypic synonymy in the genus *Streptosporangium*, all publicly available genomic sequences for species with validly published names were subjected to ANI analysis (Table S1, available in the online Supplementary Material) as described above. Only strains GW-12028^T^, IFO 13990^T^ and DSM 43181^T^ shared ANI of >96.0%. The relatedness of strains GW-12028^T^, IFO 13990^T^ and DSM 43181^T^ was also investigated using the Type (Strain) Genome Server [18]. Based on a dDDH value of 81.5% (Table 1), it was determined that the genomes of strains GW-12028^T^ and IFO 13990^T^ belong to the same species, as the currently accepted species cut-off boundary of >70.0% [20] was exceeded. A value of 66.2% was obtained upon comparison with *S. pseudovulgare* DSM 43181^T^. For reasons outlined by Meier-Kolthoff and Göker [21], dDDH was deemed the best method for delineating prokaryotic species. Finally, a phylogenomic tree was reconstructed using the Codon Tree Method [22] (Fig. 2). This method again demonstrated an extremely close relationship between *S. sandarakinum* GW-12028^T^ and *S. nondiastaticum* IFO 13990^T^, while separating them from other *Streptosporangium* species, including *S. pseudovulgare* DSM 43181^T^.

**Fig. 2.**
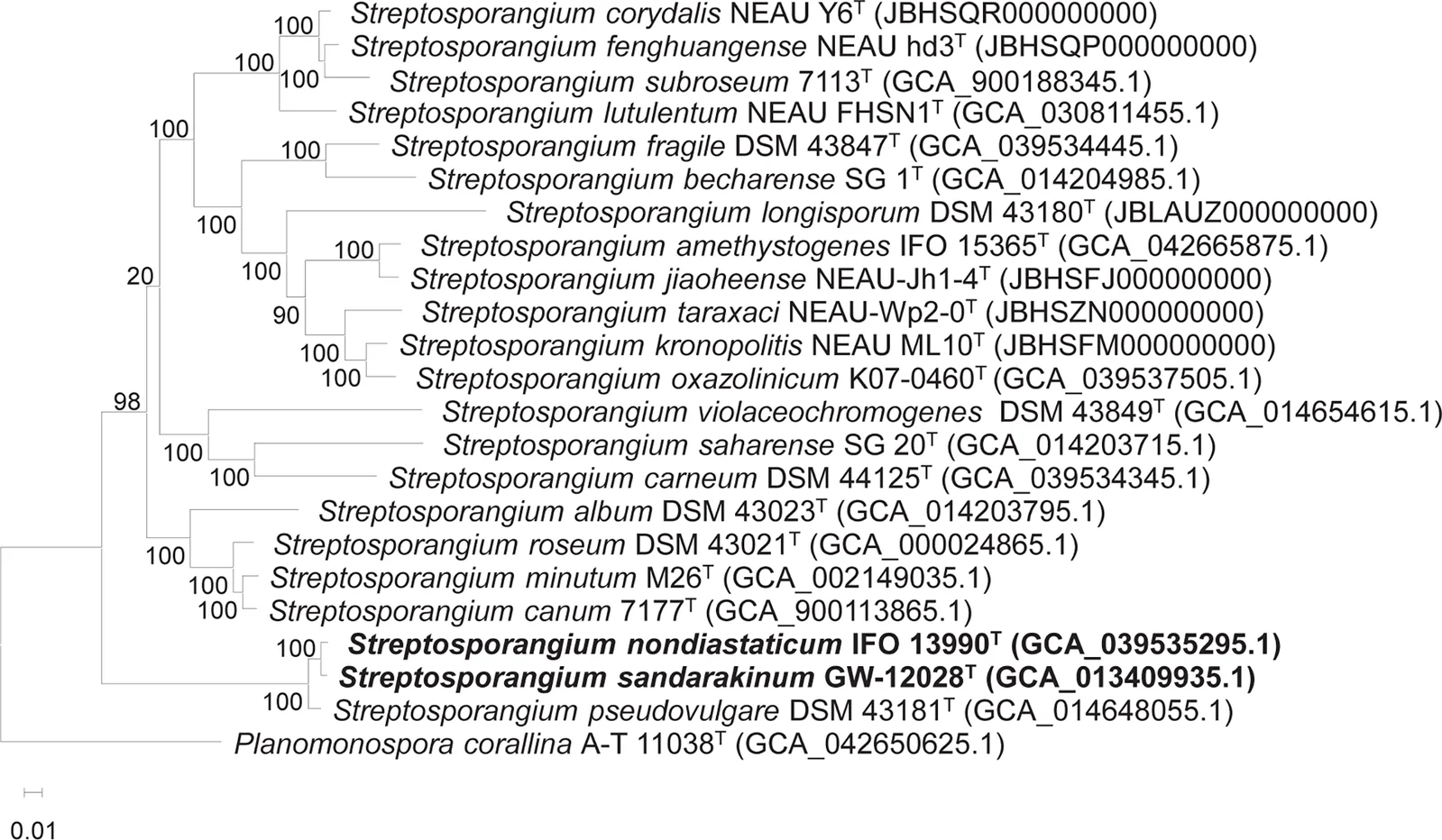
A phylogenomic tree based on the genomes of the core-genome phylogenomic tree using a concatenated alignment of 100 single-copy genes showing the relationship among *S. sandarakinum* GW-12028^T^ and *S. nondiastaticum* IFO 13990^T^ and their nearest phylogenetic relatives. The tree was constructed using Codon Tree. Database accession numbers are given in parentheses. Bar indicates the mean number of substitutions per site, 0.01.

The biochemical and physiological traits of *S. sandarakinum* GW-12028^T^ and *S. nondiastaticum* IFO 13990^T^, as reported in previous studies [7, 23], have demonstrated high levels of similarity. This is expected, as the two strains were isolated from soil and share a high degree of genetic similarity (Table 1). The observed phenotypic and metabolic incongruities are likely due to intraspecific phenotypic variability and differences in assay sensitivities.

In conclusion, we suggest that *S. sandarakinum* GW-12028^T^ and *S. nondiastaticum* IFO 13990^T^ belong to the same species based on their overall genomic relatedness indices (Table 1). This is supported by 16S rRNA gene phylogenetic trees, core-genome phylogenomic trees (Figs 1 and 2) and the highly similar physiological characteristics reported. As per the priority of prokaryotic names governed by the International Code of Nomenclature of Prokaryotes [24], when species are united, the earliest validly published name is used for the union. Therefore, *S. sandarakinum* Kämpfer *et al*. 2013 is proposed to be a later heterotypic synonym of *S. nondiastaticum* Nonomura and Ohara 1969.

## Emended description of *Streptosporangium nondiastaticum* Nonomura and Ohara 1969 (Approved Lists 1980)

The description is as before [7, 8, 23] with the following modifications.

The type strain is IFO 13990^T^ (ATCC 27101^T^=DSM 43848^T^=JCM 3114^T^=KCTC 9434^T^=NBRC 13990^T^=VKM Ac-1299), and strain GW-12028 (DSM 45763=LMG 27062) is an additional strain. The additional strain of *S. nondiastaticum* contains the major quinones, MK-9, MK-9(H_2_) and MK-9(H_4_). The utilization of carbon sources, sorbitol and melibiose, as well as cellulose degradation and nitrate reduction to nitrite, is variable.

The DNA G+C content (mol%) is 72.5.

## Supplementary material

10.1099/ijsem.0.007359 Table S1.

## Abbreviations

ANI: average nucleotide identity
dDDH: digital DNA–DNA hybridization
MK-9: menaquinone-9
ML: maximum-likelihood
